# Structural Insights into Substrate Specificity in Variants of *N*-Acetylneuraminic Acid Lyase Produced by Directed Evolution

**DOI:** 10.1016/j.jmb.2010.08.008

**Published:** 2010-11-19

**Authors:** Ivan Campeotto, Amanda H. Bolt, Thomas A. Harman, Caitriona Dennis, Chi H. Trinh, Simon E.V. Phillips, Adam Nelson, Arwen R. Pearson, Alan Berry

**Affiliations:** 1 Astbury Center for Structural Molecular Biology, Garstang Building, University of Leeds, Leeds LS2 9JT, UK; 2 School of Chemistry, University of Leeds, Leeds LS2 9JT, UK

**Keywords:** NAL, *N*-acetylneuraminic acid lyase, ManNAc, *N*-acetylmannosamine, Neu5Ac, *N*-acetylneuraminic acid, DPAH, (5*R*,6*R*)-7-(dipropylamino)-4,5,6-trihydroxy-2,7-dioxoheptanoic acid, DHOB, (2*R*,3*S*)-2,3-dihydroxy-4-oxo-*N*,*N*-dipropylbutanamide, THB, (2*R*,3*R*)-2,3,4-trihydroxy-*N*,*N*-dipropylbutanamide, PDB, Protein Data Bank, PEG, polyethylene glycol, directed evolution, X-ray crystallography, *N*-acetylneuraminic acid lyase, substrate specificity, protein engineering

## Abstract

The substrate specificity of *Escherichia coli N*-acetylneuraminic acid lyase was previously switched from the natural condensation of pyruvate with *N*-acetylmannosamine, yielding *N*-acetylneuraminic acid, to the aldol condensation generating *N*-alkylcarboxamide analogues of *N*-acetylneuraminic acid. This was achieved by a single mutation of Glu192 to Asn. In order to analyze the structural changes involved and to more fully understand the basis of this switch in specificity, we have isolated all 20 variants of the enzyme at position 192 and determined the activities with a range of substrates. We have also determined five high-resolution crystal structures: the structures of wild-type *E. coli N*-acetylneuraminic acid lyase in the presence and in the absence of pyruvate, the structures of the E192N variant in the presence and in the absence of pyruvate, and the structure of the E192N variant in the presence of pyruvate and a competitive inhibitor (2*R*,3*R*)-2,3,4-trihydroxy-*N*,*N*-dipropylbutanamide. All structures were solved in space group *P*2_1_ at resolutions ranging from 1.65 Å to 2.2 Å. A comparison of these structures, in combination with the specificity profiles of the variants, reveals subtle differences that explain the details of the specificity changes. This work demonstrates the subtleties of enzyme–substrate interactions and the importance of determining the structures of enzymes produced by directed evolution, where the specificity determinants may change from one substrate to another.

## Graphical abstract

**Figure:**
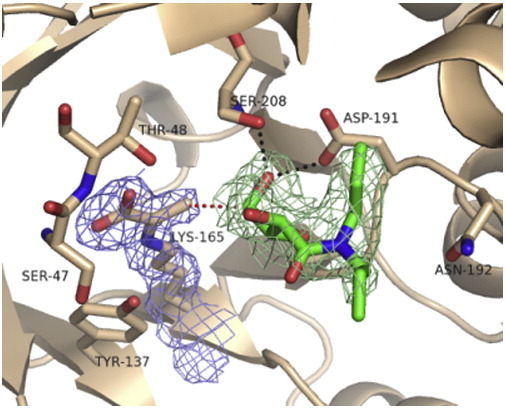

## Introduction

**Fig. 1:**
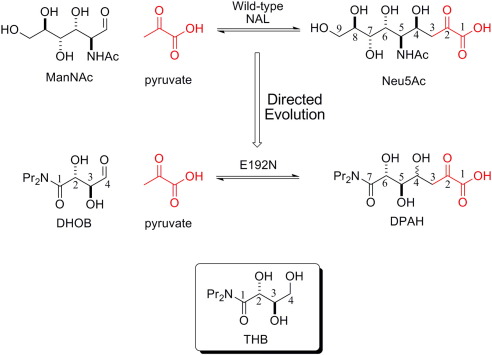
Wild-type NAL catalyzes the reaction of pyruvate (red) with ManNAc. The specificity of this reaction was altered^1^ to catalyze the reaction of pyruvate with DHOB by the mutation of Glu192 to Asn. Inset: The structure of the competitive inhibitor THB. Carbon atoms are numbered for Neu5Ac, DPAH, DHOB, and THB. Note that, upon aldol condensation, C1, C2, C3, and C4 of DHOB become C7, C6, C5, and C4 of DPAH, respectively.

**Fig. 2:**
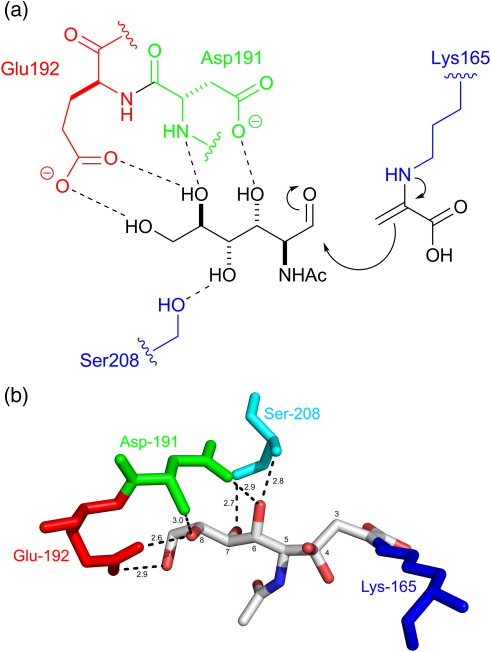
(a) Schematic showing the interactions between residues Asp191, Glu192, and Ser208, and ManNAc. A Schiff base is formed between pyruvate and Lys165. (b) X-ray crystal structure of NAL from *H. influenzae* in complex with the substrate analogue 4-oxo-sialic acid (PDB code 1F7B). The ketone at C4 is hydrated. The covalent bond with residue Lys165 is visible, and distances between the heteroatoms in residues Glu192, Asp191, and Ser208, and the heteroatoms in 4-oxo-sialic acid are highlighted.

*N*-Acetylneuraminic acid lyase (NAL; EC 4.1.3.3, otherwise known as *N*-acetylneuraminic acid aldolase or sialic acid aldolase) catalyzes the reversible aldol condensation of *N*-acetylmannosamine (ManNAc) with pyruvate to yield the most abundant sialic acid *N*-acetylneuraminic acid (Neu5Ac) (Fig. 1). Sialic acids are essential components of complex carbohydrates that play pivotal roles as recognition signals in a variety of biological processes.^2^ Such molecular recognition events are well exemplified in host–pathogen and host–parasite interactions where the oligosaccharide is often required for the invasion, infectivity, and survival of the invading organism in the host (for a review of the roles of sialic acids, see Rosenberg^3^). Sialic acid mimetics can, therefore, be important chemotherapeutic agents^4,5^ (e.g., the anti-influenza drugs Relenza and Tamiflu).^6^ NAL has been used in a number of syntheses of such analogues;^7^ however, its synthetic utility has been limited by its substrate specificity (e.g., two-carbon and three-carbon aldehyde acceptors are very poor substrates)^8^ and by the poor facial stereoselectivity of wild-type NAL^9^ and other members of the NAL superfamily during carbon–carbon bond formation.^10^ To overcome these problems, we have used structure-guided protein engineering and directed evolution of the *Escherichia coli* enzyme to create a pair of complementary biocatalysts to synthesize either possible diastereoisomeric aldol product^11^ or to tailor the enzyme for the parallel synthesis of sialic acid mimetics.^1,12^ We have reported that the engineered NAL variant E192N catalyzes the reversible cleavage of *N*-dialkylcarboxamide analogues of Neu5Ac—such as (5*R*,6*R*)-7-(dipropylamino)-4,5,6-trihydroxy-2,7-dioxoheptanoic acid (DPAH) into pyruvate and (2*R*,3*S*)-2,3-dihydroxy-4-oxo-*N*,*N*-dipropylbutanamide (DHOB) (Fig. 1)—five times more effectively that the wild-type enzyme catalyzes the cleavage of Neu5Ac.^1^ The residue responsible for this alteration of specificity at position 192 lies at one end of the active-site cleft opposite the active-site catalytic residue Lys165, which forms a Schiff base with the substrate as part of the catalytic cycle (Fig. 2a). In order to probe the mechanism by which alteration of the residue at position 192 affects substrate specificity, we have undertaken an analysis of the activity of all 20 variants of NAL at position 192 along with an X-ray structural analysis of the E192N enzyme in the absence and in the presence of a competitive inhibitor, (2*R*,3*R*)-2,3,4-trihydroxy-*N*,*N*-dipropylbutanamide (THB) (Fig. 1). These studies have provided new insights into the binding of substrates to the wild-type and variant enzymes and suggest the structural basis for the discrimination of substrates by the E192X variants.

## Results and Discussion

The importance of various interactions between wild-type NAL and different substrates or substrate analogues has been revealed by an analysis of the substrate specificity of wild-type NAL^13^ and by X-ray crystal structures. In particular, the structures of *E. coli* NAL in the absence of any substrate,^14^ the hydroxypyruvate or sodium-borohydride-reduced pyruvate complexes,^15^ and *Haemophilus influenzae* NAL in the absence of substrate or in the presence of sialic acid alditol (Neu5Ac2ol), 4-deoxysialic acid (4d-Neu5Ac), or 4-oxo-sialic acid (4oxo-Neu5Ac)^16^ have revealed that the residues at positions 191, 192, and 208 (*E. coli* numbering is used throughout this article) interact strongly with the hydroxyl groups at C7, C8, and C9 of Neu5Ac (Fig. 2). Saturation mutagenesis of these residues in the *E. coli* enzyme^1,12^ allowed us to identify residue 192 as crucial in switching the substrate specificity from Neu5Ac to DPAH.^1^

### Activity profile of NAL variants at position 192

**Fig. 3:**
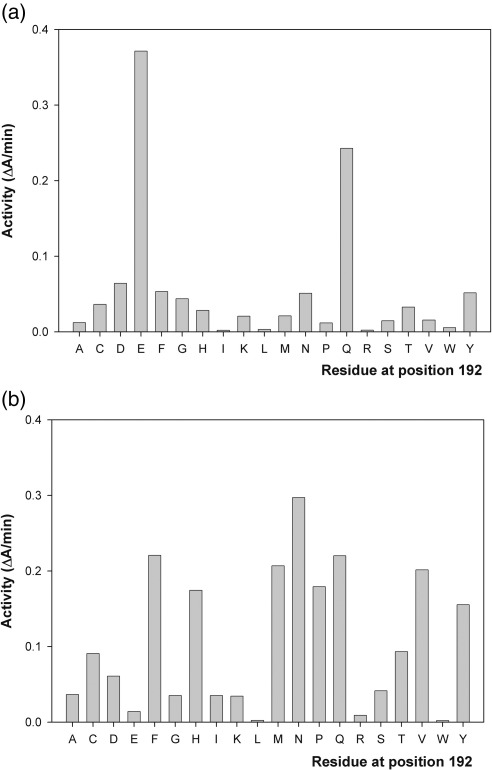
Activity of NAL variants isolated and purified from the E192X saturation mutagenesis library with (a) Neu5Ac (0.5 mM) and (a) DPAH (0.5 mM). Each assay was carried out as described in Materials and Methods and contained ∼ 0.03 mg of purified enzyme.

In order to determine the relationship between the amino acid at position 192 and the activity with various compounds, we sequenced 96 clones from an E192X saturation mutagenesis library.^1^ This allowed us to identify 15 of the 20 possible variants of NAL at position 192. The remaining variants (E192A, E192F, E192H, E192Q, and E192Y) were then constructed by site-directed mutagenesis. All variants of the E192X library were expressed in *E. coli* as hexa-His-tagged proteins and purified to homogeneity on Ni-NTA resin. The activity of each enzyme for the cleavage of either Neu5Ac or DPAH was determined at a single low concentration of substrate (0.5 mM) so that the enzymes were unlikely to be saturated (the *K*_m_ of the wild-type enzyme for Neu5Ac is ∼ 4 mM^1^), and the observed rates may be an approximation of *k*_cat_/*K*_m_ (Fig. 3). These results show that the activity towards Neu5Ac is highest for the wild-type enzyme (E192), and only the E192Q variant shows significant activity towards this substrate. The steady-state kinetic parameters *k*_cat_ and *K*_m_ were then determined for the cleavage of Neu5Ac with several NAL variants, as shown in Table 1. These results show that correct interaction between Neu5Ac and residue 192 is extremely important for binding and catalysis. In the X-ray crystal structure of *H. influenzae* NAL in complex with 4-oxo-Neu5Ac,^16^ residue Glu192 hydrogen bonds with the diol at C8 and C9 of the sialic acid analogue (Fig. 2), and it is likely that the glutamine introduced in E192Q mimics this interaction so well that the kinetic parameters of the E192Q enzyme with Neu5Ac (Table 1) are not significantly altered with respect to wild type. This profile of activity, with only E192 and E192Q producing significant activity, explains the distribution of activities towards Neu5Ac found during the initial isolation of a NAL variant capable of turning over the dipropylamide DPAH.^1^ Interestingly, maintaining the hydrogen-bonding capacity at position 192 while reducing the length of the side chain (e.g., in the E192D or E192N variants) severely reduced the activity (*k*_cat_/*K*_m_) to less than 15% of the wild-type value with Neu5Ac (Table 1). This is presumably because the substrate, in its extended conformation, cannot span from the introduced aspartate or asparagine to lysine 165, where it has to form a Schiff base. This finding is also in line with previous studies that have shown that short carbon chain analogues of Neu5Ac are not substrates for the wild-type enzyme.^8^

The activity profile of the E192X library with DPAH differs dramatically from that with Neu5Ac, as many more of the variants (e.g., E192F, E192H, E192M, E192N, E192Q, E192P, and E192V) show significant activity with the new substrate (Fig. 3). The steady-state kinetic parameters of these enzymes (Table 1) confirmed that the E192N enzyme had the highest activity for DPAH. It is interesting to note that E192Q shows a high level of activity with DPAH, making this enzyme the most promiscuous in terms of substrate specificity. All of the active variants showed *k*_cat_ values generally similar to those of the wild-type enzyme, and changes in the specificity constant *k*_cat_/*K*_m_ were all brought about by changes in *K*_m_ for the substrate (Table 1), implying alterations in the ability of the enzyme to recognize and bind the substrate depending on the diversity of functionality at position 192. The range of active functionalities at position 192 suggests that the interaction with the substrate in these enzymes is less specific than the interaction between E192 and Neu5Ac, and that a number of residues are able to make interactions with DPAH to promote catalysis. Interestingly, some of the active variants have large hydrophobic side chains at position 192 (E192F, E192Y, and E192M), and we reasoned that these side chains may provide a hydrophobic environment with which the propyl groups of DPAH are able to interact in a favorable manner. In order to gain further insights into the mechanism of substrate specificity in the E192 variants, we embarked on an X-ray crystallographic analysis of the ‘best’ enzyme for dipropylamide cleavage (namely E192N) and on a structural modeling of the possible binding modes of interaction of DPAH with other E192 variants.

### Crystallographic results

NAL has a (β/α)_8_ barrel structure of the type first found in triose phosphate isomerase,^17^ and crystal structures of *E. coli* NAL in the absence of ligands [Protein Data Bank (PDB) code 1NAL],^14^ in complex with hydroxypyruvate (PDB code 1FDY), or trapped by sodium borohydride in the presence of pyruvate (PDB code 1FDZ)^15^ were already available in the PDB database. In addition, the structure of an L142R mutant of *E. coli* NAL, which was evolved *in vitro* to have increased dihydrodipicolinate synthase activity, was also available in complex with hydroxypyruvate (PDB code 1HL2).^18^ We reasoned that a structural understanding of the features that caused the change in the specificity of the E192N variant away from the reaction of pyruvate with ManNAc towards that with DHOB would only be forthcoming if we could solve the structure of NAL in the presence of substrates or substrate analogues that mimicked the full-length substrate. However, with the exception of the L142R mutant NAL structure,^18^ *E. coli* NAL crystals had been obtained after crystal growth in high-salt concentrations, resulting in the presence of a sulfate ion in the catalytic pocket that precluded co-crystallization with ligands larger than pyruvate.^14,16^ We therefore identified new conditions for the crystallization of *E. coli* NAL (in space group *P*2_1_) that avoided the use of sulfate ions and allowed us to solve the structures of both the wild type and the E192N variant in the presence and in the absence of a number of ligands.

**Fig. 4:**
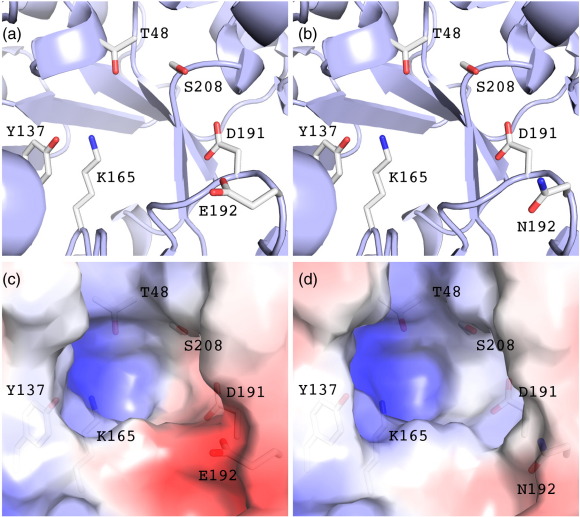
Close-up view of the active-site cleft of wild-type NAL (a) and the E192N variant (b). The backbone is shown as a gray cartoon, and active-site residues are shown as sticks colored by atom type. (c) Wild type and (d) E192N are the same view and show the solvent-accessible surface colored by electrostatic potential (calculated in PyMOL; blue, positive; red, negative) to illustrate the change in surface potential as a result of glutamate-to-asparagine mutation, as well as the position of residue 192 at the entrance to the active-site cleft.

The crystal structures of wild-type *E. coli* NAL and the E192N variant were solved at 2.2 Å and 1.8 Å, respectively (Table 2), and show a tertiary structure identical to that previously observed.^14^ The overall RMSD between the C^α^ atoms in the wild-type structure and the C^α^ atoms in the E192N structure is 0.3 Å. The only significant differences are the orientation of Asn192 compared to that of the wild-type Glu192 and the resulting changes in surface potential at the entrance of the catalytic pocket when the negatively charged glutamate is replaced (Fig. 4).

**Fig. 5:**
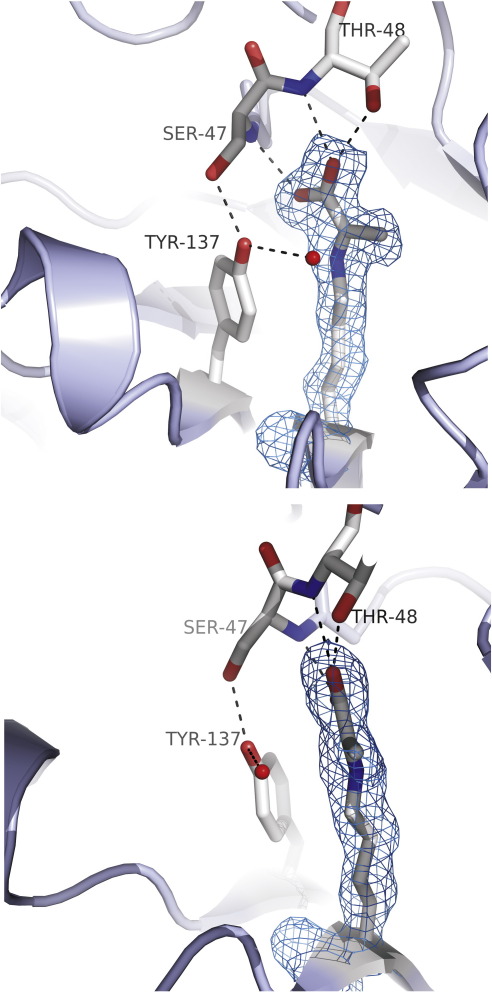
Active site of the wild-type NAL–pyruvate complex. Two views of the active site of the wild-type NAL–pyruvate complex showing the interactions of the pyruvate enamine with the enzyme. Pyruvate is covalently linked to Lys165 and makes hydrogen bonds with Ser47 and Thr48. The hydrogen-bonding network to Tyr137 and a conserved water molecule are also shown. The lower panel shows a rotation of the molecule to display the planar nature of the 2*F*_o_ − *F*_c_ electron density (contoured at 1 RMSD) of pyruvate–enzyme bonding confirming the enamine structure for this intermediate.

The structures of complexes with pyruvate were also obtained (to 1.65 Å resolution for the wild-type enzyme and to 1.8 Å resolution for E192N; Table 2). As expected, pyruvate was covalently linked to Lys165, and the planarity of electron density is consistent with the formation of the natural pyruvate enamine intermediate (Figs. 2a and 5). We have previously reported the structure of the E192N variant, in space group *P*2_1_2_1_2_1_, in complex with pyruvate.^19^ The interactions made by pyruvate in that structure and in the *P*2_1_ structures of the wild-type–pyruvate and E192N–pyruvate complexes reported here are identical. The carboxylate group of pyruvate forms hydrogen bonds with the backbone amides of Ser47 and Thr48 and also with the side-chain OH of Thr48 (Fig. 5), as previously found for pyruvate and hydroxypyruvate^15^ and as is typical of a number of related pyruvate-dependent aldolases.^16^ Comparison with the apo-NAL structures indicates that there are no significant changes in the active site upon pyruvate binding; the only difference in the two structures appears to be a further disordering of the C-terminal residues in a remote location from the catalytic pocket.

**Fig. 6:**
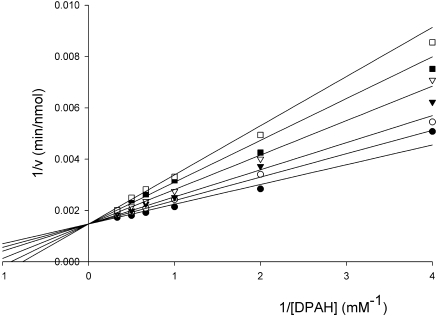
Lineweaver–Burk plot of the steady-state enzyme kinetics of E192N reaction in the absence and in the presence of the inhibitor THB: (●) absence of THB; (○) 0.25 mM THB; (▼) 0.5 mM THB; (▽) 1 mM THB; (■) 1.5 mM THB; (□) 2 mM THB. The initial rates of reaction were measured using the standard enzyme assay, and kinetic parameters were determined by fitting all data to the rate equation for a single-substrate reaction in the presence of a competitive inhibitor to measure the *K*_i_ for THB.

Soaking the E192N variant with the substrate DPAH in order to trap a DPAH–NAL complex was unsuccessful due to the rapid rate of the cleavage reaction. As a result of this, only density for the cleavage product (pyruvate covalently linked to Lys165) was observed, and this structure was identical with the E192N–pyruvate complex described above (data not shown). To overcome this problem, we designed an analogue of the acceptor DHOB (Fig. 1) in the E192N-catalyzed reaction. This compound, THB (Fig. 1), mimics the substrate DHOB, but the reactive aldehyde has been reduced to an alcohol, preventing nucleophilic attack of pyruvate enamine. THB was synthesized,^20^ and steady-state enzyme kinetics showed that it is a competitive inhibitor of the E192N NAL reaction (Fig. 6). The *K*_i_ of THB for the reaction was measured as 1.3 mM.

**Fig. 7:**
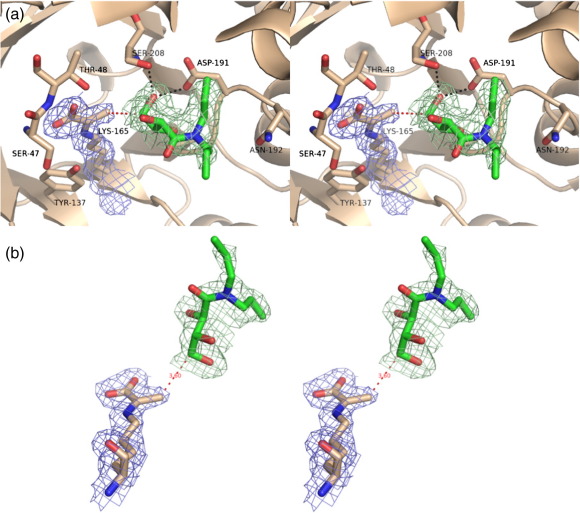
X-ray crystal structure of the E192N NAL–pyruvate–THB complex. (a) The active site of the E192N NAL enzyme in the presence of pyruvate and the competitive inhibitor THB. The green net shows a THB electron density omit map contoured at an RMSD of 1.5. Interactions of pyruvate enamine with Ser47 and Thr48, as well as the interactions of THB with Ser208 and Asp191, are also shown. (b) Details of Lys165–pyruvate enamine and THB. The green net shows the THB omit map as described above. 2*F*_o_ − *F*_c_ electron density contoured at an RMSD of 1.0 is shown as a blue net for the Lys165–pyruvate Schiff base. The distance (3.6 Å) between the pyruvate methyl group and the C4 of THB (equivalent to the new carbon–carbon bond formed between the substrate acceptor and the donor) is shown as a red dotted line.

**Fig. 8:**
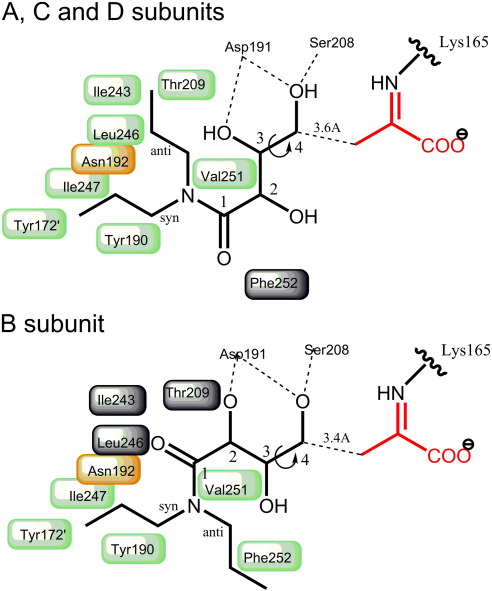
Schematic representation of THB binding to E192N NAL. Top: Binding of THB in subunits A, C, and D. THB (black lines) binds in the active-site pocket, with its C4 positioned 3.6 Å from the C3 of pyruvate (red) bound as a Schiff base to Lys165. Ser208 and Asp191 make H-bonds with the C4–OH and C3–OH groups. Asn192 (orange shaded box) lies between the *syn*-propyl arm and the *anti*-propyl arm. The hydrophobic sites (green shaded boxes) for these arms are formed from Tyr190, Ile247, and Tyr172′ (*syn*), and a deep pocket is formed by Leu246, Ile243, Thr209, and Val251 (*anti*). In this binding mode, Phe252 (gray shaded box) appears to have little binding role. Bottom: Binding of THB in subunit B. Rotation of THB around the C3–C4 bond compared with binding in subunits A, C, and D positions THB such that Ser208 and Asp191 now H-bond to the C4–OH and C2–OH groups and Asn192 lies between the *syn* arm and the C1 carbonyl group. This rotation positions the *syn* arm in the same hydrophobic binding site (green boxes); however, in this orientation, the *anti* arm is bound against Val251 and Phe252 (green boxes), and the deep hydrophobic pocket (gray boxes) is empty. Note that Val251 lies in a position where it can form part of the deep hydrophobic pocket and the site for the *anti* arm in subunit B.

The structure of the E192N complex with pyruvate and THB was solved at 2.05 Å resolution and, overall, appeared identical with the structure of E192N in complex with pyruvate, with additional electron density only observed in the active site. This strong electron density corresponds to the pyruvate enamine with residue Lys165. The planarity of this electron density confirms the expected sp^2^ hybridization for the carbon–carbon double bond of the enamine intermediate. The noncovalent interactions of pyruvate with the protein are identical with those observed for the wild-type–pyruvate and E192N–pyruvate complexes. The *F*_o _− *F*_c_ electron density map also revealed a further strong positive electron density in the enzyme active site. The inhibitor THB fits well into this density, with the exception of the terminal ends of the two propyl groups, likely due to the freedom of rotation of these groups (Fig. 7). There was some variation in the quality of the electron density and in the final modeled orientation for THB in the four crystallographically independent subunits of the tetramer, with subunit D showing the most well-defined THB density. The electron density in subunit B was more consistent with a slightly different binding mode for THB in comparison to subunits A, C, and D (Fig. 8) (see the text below and Table S1).

In all four subunits, H-bonds are formed between the C4 hydroxyl group of THB and the backbone amide of Ser208 and the side chain of Asp191 (Fig. 8). In addition, the backbone carbonyl group of Gly189 makes H-bonds with the THB [with the C3 hydroxyl group in subunits A, C, and D, and with the C2 hydroxyl in subunit B (equivalent to C5 and C6 of DPAH)]. These interactions result in the more polar portion of the inhibitor (containing the three hydroxyl groups) being bound at the base of the catalytic pocket next to the pyruvate (Figs. 7 and 8). During enzyme catalysis, a new carbon–carbon bond is formed between C3 from the pyruvate enamine and the C4 from the aldehyde acceptor. In the enzyme–pyruvate–THB complex structure, this bond cannot be formed, as THB is unable to undergo nucleophilic attack by the enamine C3. Nonetheless, the C3 of the pyruvate enamine lies only ∼ 3.5 Å from the C4 of THB, compatible with these two atoms being in van der Waals contact. This distance compares well with the separation of the substrate aldol donor and acceptor molecules (3.3–4.4 Å) found in the X-ray structure of a different aldolase, the *Mycobacterium tuberculosis* fructose bisphosphate aldolase,^21^ and therefore represents the Michaelis entry (or exit) complex in which the two substrates are ideally placed, ready for the nucleophilic attack of the enamine on the aldehyde of the acceptor substrate.

The structure of E192N, in complex with pyruvate and THB, reveals the structural basis of the discrimination between substrates manifested by wild-type and E192N NAL. In the wild-type enzyme, residue Glu192 is sandwiched between Tyr190 of the same subunit and Tyr172 of a neighboring subunit. In this position, its carboxylic acid group points towards the active-site cleft, perfectly positioned to form strong hydrogen bonds with the O8 and O9 of the glycerol moiety of the incoming substrate Neu5Ac (or the equivalent O5 and O6 of ManNAc). In contrast, in the E192N variant, the amide of Asn192 points out of the active-site cleft (a position disfavored for glutamate due to unfavorable contacts with the neighboring hydrophobic residues Tyr172 and Ile243), and this conformation produces a larger hydrophobic binding surface that is ideal for interaction with THB. The two conformations of THB (found in subunits A, C, and D compared with subunit B; Fig. 8) occur because of a rotation of the main-carbon chain of THB around the C4–C3 bond. This places the two propyl arms in different positions in the two conformations. In subunits A, C, and D, the *syn* arm of the dipropylamide lies against the hydrophobic surface of Tyr190, Ile247, and Tyr172 (this latter residue is from a neighboring subunit), while the *anti* arm lies in a deep hydrophobic pocket composed of Ile243, Thr209, Val251, and Leu246. In this binding conformation, the mutated residue at position 192 lies at the bifurcation of the two propyl arms of THB. Modeling of a glutamate residue at position 192 shows that the surface of the wild-type enzyme would be more polar in this important binding region and that there would also be steric clashes between the enzyme carboxylate and the C1 and C2 atoms of the dipropylamide arms. In subunit B, the other conformation of bound THB places the *syn* arm of the inhibitor in the same region as in subunits A, C, and D, on the surface of Tyr190 and Ile247; however, in this case, the hydrophobic pocket is empty, with the *anti* arm instead lying against another hydrophobic part of the surface formed from residues Val251 and Phe252. In this case, the introduced Asn192 lies between the *syn*-propyl arm and the amide carbonyl group. The modeled position of the wild-type glutamate residue again shows steric clashes of the Glu192 carboxylate, this time with both the *syn*-propyl and the amide O atom. With the assumption that the position and orientations of the bound competitive inhibitor THB accurately reflect the binding of the substrate DPAH, the structural basis of discrimination against DPAH binding to the wild-type enzyme and in favor of binding to the E192N variant becomes clear. The larger glutamate at position 192 in the wild-type enzyme prevents DPAH binding not only through steric clashes with the substrate, which cannot be relieved because of the planarity of the substrate, but also through the negative charge of Glu192 making unfavorable interactions with the hydrophobic parts of DPAH. An asparagine at position 192, on the other hand, provides an uncharged surface with no steric interference with DPAH binding. These results also explain the substrate activity profile found previously for the E192N variant.^12^ Dialkyl substituents, including dibutylamides, are well accepted by the E192N enzyme, and we would expect that the *syn* arm of each amide would bind similarly to that of THB, while the hydrophobic pocket formed from Ile243, Thr209, Val251, and Leu246 described above is deep enough to accommodate the longer butyl group. We suggest that other active substrates, including pyrrolidinyl amide, morpholinyl amide, and piperidinyl amide,^1,12^ interact with the E192N variant through the extensive hydrophobic surface generated by removing the charge from position 192, and this explains why the more hydrophobic cyclic substrates are preferred.

### Understanding the role of residue 192 in substrate selectivity

Catalysis of the aldol condensation/cleavage of the natural substrates of NAL is supported by a narrow range of amino acids at position 192 (E and Q; Fig. 3a) characterized by their polarity and size, as described above. In contrast, the reaction with DPAH and other dialkyl derivatives tolerates a much wider range of side-chain size and polarity at this position (Fig. 3b), with E192Q showing high levels of activity with both natural and synthetic substrate classes. Our structural investigations of the E192N enzyme and, in particular, its complex with the inhibitor THB have enabled us to propose a general structural basis for these findings. The successful binding and catalysis of the reaction with dialkylamides clearly require a suitably sized hydrophobic binding site. Very large residues at position 192 (e.g., E192W) or charged residues (E192R, E192K, E192, and E192D) would interfere with substrate binding by breaking up the hydrophobic binding surface required, and this is reflected in their low activity (Fig. 3b). On the other hand, we suggest that small residues at position 192 (E192G, E192A, and E192S) do not provide enough surface for interaction with the substrate, explaining their lack of activity. Of more interest is how larger residues at position 192 (e.g., E192F, E192Y, E192Q, E192M, E192V, and E192P) are still able to promote reasonable levels of activity when we might have expected these large residues to clash with the alkyl arms of the substrate. However, *in silico* mutagenesis of residue 192 revealed a number of side-chain conformations that allow packing of the mutated side chain into the overall protein structure without significant clashes with the position of the THB bound in our crystal structure. For example, both Tyr192 and Phe192 can pack in a hydrophobic site between residues Tyr172 and Tyr190, providing a suitable hydrophobic surface for THB binding without steric clashes. Similarly, Met192 can lie in this region with the terminal methyl group rotated away from the substrate to provide room for THB binding, while smaller hydrophobic residues such as valine and proline can also fit into the folded chain at position 192 without serious distortions to the backbone of the protein and without interfering with the binding of THB and hence of the dialkylamide substrates. A similar analysis reveals the basis of the promiscuity of the E192Q enzyme (Fig. 3a and b). If modeled in the same conformation as the native glutamate, the introduced glutamine is able to form H-bonds with the O8 and O9 hydroxyl groups of the natural substrate Neu5Ac. However, this conformation of the glutamine would cause rejection of DPAH as a substrate because of steric interference. On the other hand, an alternative conformation of the neutral glutamine side chain, in which it can pack between the two active-site tyrosine residues in a conformation disfavored for the negatively charged glutamate residue, is possible. In this conformation, it provides a hydrophobic surface with no steric problems for DPAH binding. In this way, the E192Q enzyme is able to provide alternative modes of substrate binding that account for its promiscuity.

## Conclusions

The directed evolution approach for the construction of new substrate specificities does not require any previous structural or mechanistic knowledge of catalysis in the target enzyme, and sequence analysis of the resultant successful variants selected often contains unexpected residues at unexpected positions to produce the desired activity. However, a full understanding of the mechanisms underlying the changes in specificity is required if we are to learn how to rationally produce new activities, and reports of the structures of enzymes with altered substrate specificity created by directed evolution are appearing. These have revealed that altered substrate specificity can be brought about in a number of ways. For example, in aspartate aminotransferase, changes in residues involved in direct binding to the substrate^22^ or conformational changes in the enzyme brought about by mutations often at a distance of > 10 Å from the active site^23^ have been demonstrated to affect specificity. Similarly, the promotion of a more compact conformation of the acyltransferase LovD brought about by mutations was found to be important in creating a simvastatin synthase from this template.^24^ Finally, significant changes in the size and conformation of active-site residues resulting in the reshaping of the active site were considered a major determinant of the specificity switches found in evolved enantioselective epoxide hydrolase.^25^ Here, by combining substrate specificity profiles with X-ray crystal structure determination, we have been able to dissect the way that the residue present at position 192 of NAL can affect substrate specificity. The change from Glu192 to Asn192 to switch the specificity from Neu5Ac to DPAH was not a change we would have rationally chosen to move from a triol-containing substrate to the more hydrophobic and bulkier dipropylamide. Our results have shown that when the mutations at position 192 provide an extensive hydrophobic surface capable of binding dipropyl-containing substrates, there are a number of conformations of the substrate that result in activity. However, if there is too much space between the enzyme and the substrate (e.g., with small or hydrophilic residues at position 192), then the H-bonding at the other end of the substrate is not sufficient to provide enough binding energy to hold the substrate for catalysis to occur. This accords with the lack of enzyme activity for ‘short’ two-carbon or three-carbon aldehyde substrates.^8^ These results point the way towards engineering NAL variants with activity for such substrates, perhaps by reducing the size of the active site around the aldehyde substrate binding pocket, and highlight the need for future structural studies of evolved enzymes.

## Materials and Methods

### Expression and purification of NAL

*E. coli* BL21-competent cells were transformed with the plasmid pK*nan*A harboring the wild-type NAL gene or the E192X NAL library members, as previously described.^1^ Cells were grown in 5 mL of 2YT medium containing 50 μg mL^− 1^ ampicillin and inoculated in 2 L of 2YT medium containing 50 μg mL^− 1^ ampicillin and 0.4 mM IPTG for 16 h at 30 °C. Cells were lysed in a Cell Disruption Systems apparatus at 19,000 psi in 50 mM potassium phosphate buffer (pH 7.5) containing 300 mM NaCl supplemented with protease cocktail inhibitor (Roche). NAL was bound to Qiagen Ni-NTA resin equilibrated in the same buffer containing 30 mM imidazole and was eluted at 500 mM imidazole. Purified NAL was dialyzed overnight against 50 mM Tris–HCl and 150 mM NaCl (pH 7.5). Further purification was achieved by size-exclusion chromatography using an S-200 gel-filtration column (Amersham) in the same buffer before storage and concentration. Protein purity was assessed by SDS-PAGE, and molecular weight was confirmed with electrospray ionization mass spectrometry.

### Enzyme assay

Aldolase activity with Neu5Ac and analogues was measured using a coupled enzyme assay with lactate dehydrogenase and NADH, as previously described.^1,26^ The assay was performed at 25 °C in a 1-mL cuvette containing 50 mM Tris–HCl (pH 7.5), 0.2 mM NADH, 0.5 U of lactate dehydrogenase, and a suitable aliquot of NAL (1–100 μg). The reaction was initiated by the addition of varying concentrations of substrate (0.5–20 mM Neu5Ac and 0.1–2.5 mM DPAH; chosen to bracket the measured *K*_m_ values). The decrease in absorbance at 340 nm was recorded on a Uvikon 930 spectrophotometer as the measure of enzyme activity. One unit of aldolase activity is defined as the amount of enzyme that catalyzes the oxidation of 1 μmol min^− 1^ NADH in this system, using the molar extinction coefficient of NADH (6220 M^− 1^ cm^− 1^). The triol, THB, was synthesized as previously described,^20^ and inhibition of the NAL reaction was measured by including THB (0.25–2 mM) in the standard enzyme assay. Steady-state kinetic parameters *k*_cat_, *K*_m_, and *K*_i_ were all estimated by nonlinear regression to the appropriate rate equations.

### Construction of the E192X library

Preparation of the E192X saturation mutagenesis library was undertaken using megaprimer PCR, as previously described.^1^ DNA sequencing of 96 mutants revealed 15 of 20 different amino acids at position 192. The remaining five mutants were constructed using QuikChange site-directed mutagenesis, following the instructions provided by the manufacturer (Stratagene).

### Protein crystallization

Tests with the pre-crystallization assay kit (Hampton Research) indicated an optimum NAL concentration of 10 mg mL^− 1^. The initial conditions for crystallization were identified with an Oryx crystallization robot (Douglas Instruments) using commercial sparse matrix screens (Hampton Research). Crystals were grown at 4 °C by sitting drop diffusion at a ratio of 2 μl of protein (10 μg mL^− 1^) to 2 μl of mother liquor. Several hits were obtained, but high-salt conditions (such as 3 M (NH_4_)_2_SO_4_ or 2 M Li_2_SO_4_) were ignored on the basis of our previous studies and the data reported in the literature.^16^ The final optimized crystallization conditions were 100 mM Tris–HCl (pH 8.0–8.30), 200 mM NaCl, and 18% (wt/vol) polyethylene glycol (PEG) 3350, which yielded crystals in 7–10 days. This condition is more physiologically relevant than the published high-salt conditions^16^ and, crucially, it allowed us to soak the substrate pyruvate and the triol inhibitor in the crystals.

The crystals belonged to space group *P*2_1_ with unit cell dimensions *a* = 57 Å, *b* = 143 Å, *c* = 83 Å, and β = 109° (decimal numbers are omitted due to their variability in several structures). Occasionally, crystals also grew in space group *P*2_1_2_1_2_1_, and we have recently reported the structure of the E192N variant in complex with pyruvate^19^ in this crystal form. To form the NAL and the E192N variant pyruvate complexes, we soaked crystals in mother liquor supplemented with 100 mM sodium pyruvate (Sigma Aldrich) and 15% (vol/vol) PEG 400 (Hampton Research) for 1 min. The crystals were then sequentially transferred into mother liquid containing 20% (vol/vol) PEG 400 (with 5% increments in PEG 400 concentration) and finally soaked in mother liquor containing 25% (vol/vol) PEG 400 before being flash cooled in liquid nitrogen. In the case of the pyruvate and inhibitor complexes, the same sequential procedure was performed with the addition of 70 mM THB inhibitor in the last cryoprotection step [25% (vol/vol) PEG 400] for 2 min before flash cooling.

### Data collection and structure solution

Diffraction data for each structure were collected from single crystals on the macromolecular crystallography beamlines of Diamond Light Source (stations I02, I03, and I04) at 100 K. The data were indexed, integrated, and scaled using MOSFLM^27^ and SCALA,^28^ and further processed to generate a complete list of structure factors with *R*_free_ flags using the CCP4 suite (Table 2).^29^ Five percent of the data were randomly selected and excluded from the refinement to constitute the *R*_free_ set. We have recently reported the crystal structure of the E192N variant in complex with pyruvate in the space group *P*2_1_2_1_2_1_ (PDB code 2WKJ)^19^ at a relatively high resolution (1.45 Å). This structure was used as a molecular replacement model to solve the structure of the NAL complex with pyruvate (PDB code 2WNN) in space group *P*2_1_ using Phaser.^30^ For the other structures presented here (all belonging to space group *P*2_1_), the initial phases were calculated by combining the initial model obtained from this molecular replacement and the relevant recorded amplitudes after five cycles of rigid-body refinement in REFMAC5^31^ (treating the entire tetramer as a rigid body).

### Refinement

Refinement was performed using REFMAC5^31^ (Table 2). Isotropic *B*-factors were refined in all structures and analyzed using the CCP4 suite program BAVERAGE.^29^ Riding hydrogen atoms were included in the refinement. After each cycle of refinement, model building was carried out using Coot.^32^ Coordinates and restraint library files for the ligand THB and for a modified lysine residue, KPI (with a covalently bound pyruvoyl moiety), were obtained from the PRODRG server.^33^ The quality of the model was checked with MolProbity.^34^ The superposition of the protein structures and the calculation of their RMSDs were performed with LSQKAB.^29^

In all structures, except for that of the E192N variant in complex with pyruvate, REFMAC detected a twinning fraction between 6% and 46%, with a twinning operator − *h*, − *k*, *h* + *l*. In these structures, refinement was performed throughout using the twin refinement option in REFMAC5.

### Accession numbers

Coordinates and structure factors have been deposited in the PDB with accession numbers 2WO5 (wild type), 2WNN (wild type + pyruvate), 2WNQ (E192N), 2WNZ (E192N + pyruvate), and 2WPB (E192N + pyruvate + THB).

The following are the supplementary materials related to this article.

Table S1 Interactions between THB and the enzyme active sites of each subunit.

## Figures and Tables

**Table 1:** Steady-state kinetic parameters of wild-type and E192X variants with Neu5Ac and DPAH

Enzyme	Neu5Ac			DPAH		
	*k*_cat_ (min^− 1^)	*K*_m_ (mM)	*k*_cat_/*K*_m_ (min^− 1^ mM^− 1^)	*k*_cat_ (min^− 1^)	*K*_m_ (mM)	*k*_cat_/*K*_m_ (min^− 1^ mM^− 1^)
Wild type	260 ± 6^a^	4.4 ± 0.3^a^	59^a^	73 ± 4^a^	11 ± 2^a^	7^a^
E192N	170 ± 10^a^	38 ± 5^a^	4.4^a^	130 ± 3^a^	0.4 ± 0.04^a^	340^a^
E192Q	300 ± 20	3.5 ± 0.5	85	170 ± 4	0.7 ± 0.05	250
E192V	120 ± 8^a^	34 ± 5^a^	3.5	95 ± 4^a^	0.4 ± 0.1^a^	270^a^
E192F	160 ± 5	5.9 ± 0.4	27	88 ± 2	0.3 ± 0.02	310
E192H	120 ± 5	6.2 ± 0.7	19	38 ± 1.0	0.5 ± 0.04	80
E192M	70 ± 4	5 ± 0.8	14	70 ± 3	0.2 ± 0.03	320
E192P	25 ± 1	6 ± 0.9	4	49 ± 1	0.2 ± 0.02	260
E192D	120 ± 5	4.6 ± 0.5	26	6 ± 0.1	1.1 ± 0.05	5
E192S	50 ± 3	6 ± 0.8	8	26 ± 0.7	0.6 ± 0.04	42

The steady-state kinetic parameters of the wild-type and variant enzymes for Neu5Ac and DPAH cleavage were measured with a standard coupled enzyme assay. Kinetic parameters (± standard error of the fit) were determined by fitting the data to the Michaelis–Menten equation.

a Data taken from Williams *et al.*^1^

**Table 2:** Crystallographic data collection and refinement statistics

	Wild type	Wild type + pyruvate	E192N	E192N + pyruvate	E192N + pyuvate + THB
Diamond beamline	I02	I03	I04	I03	I03
Space group	*P*2_1_	*P*2_1_	*P*2_1_	*P*2_1_	*P*2_1_
*a*, *b*, *c* (Å),	54.8, 142.2, 84.2	54.7, 142.5, 83.6	54.6, 142.8, 84.5	56.9, 143.0, 83.9	57.0, 143.7, 83.3
β (°)	109.0	109.2	109.0	109.8	109.9
Resolution (Å)	79.56–2.20 (2.26–2.20)	79.1–1.65 (1.74–1.65)	47.60–1.80 (1.90–1.80)	79.06–1.80 (1.95–1.80)	79.31–2.05 (2.16–2.05)
*R*_merge_	0.102 (0.365)	0.060 (0.317)	0.086 (0.388)	0.070 (0.430)	0.09 (0.438)
*R*_pim_ (all *I*^+^ and *I*^−^)	0.063 (0.219)	0.038 (0.215)	0.054 (0.033)	0.043 (0.269)	0.056 (0.273)
〈*I*〉/σ〈*I*〉	8.5 (3.4)	13.5 (3.2)	8.3 (2.8)	12.0 (2.5)	8.6 (2.6)
Completeness (%)	99.9 (100)	91.2 (60.8)	98. 4 (97.2)	100 (99.9)	98.6 (96.3)
Redundancy	3.7 (3.7)	3.4 (2.9)	3.6 (3.5)	3.6 (3.5)	3.5 (3.4)
Wilson *B*-factor (Å^2^)	34.8	31.7	26.8	21.8	24.5
Number of reflections	197,621 (29,177)	449,770 (37,158)	398,910 (55,837)	391,185 (54,278)	277,866 (37,606)
Number of unique reflections	54,006 (7847)	131,694 (12,746)	110,985 (15,919)	107,356 (15,671)	78,597 (11,141)
Resolution (Å)	79.56–2.20 (2.26–2.20)	79.1–1.65 (1.69–1.65)	47.60–1.80 (1.90–1.80)	79.06–1.80 (1.95–1.80)	79.31–2.05 (2.16–2.05)
*R*-factor	0.211 (0.259)	0.209 (0.261)	0.197 (0.261)	0.188 (0.301)	0.190 (0.281)
*R*_free_	0.271 (0.356)	0.247 (0.344)	0.253 (0.391)	0.225 (0.344)	0.234 (0.337)
Twinning fraction	0.338	0.334	0.463	None	0.06
Twinning operator	− *h*, − *k*, *h* + *l*	− *h*, − *k*, *h* + *l*	− *h*, − *k*, *h* + *l*	None	− *h*, − *k*, *h* + *l*
Number of atoms					
Protein	9166	9166	9178	9292	9246
Water	169	758	316	654	540
Ligands	—	31 (PEG 400, Na^+^)	4 (Cl)	24 (PEG 400, pyruvate^a^)	60 (THB)
Average *B*-factors (Ǻ^2^)					
Protein	32.9	22.8	28.0	22.8	28.4
Waters	27.9	26.7	26.7	27.2	31.9
Ligands	—	32.4	26.3	45.8	50.1
RMSDs					
Bond lengths (Å)	0.013	0.012	0.011	0.009	0.012
Bond angles (°)	1.46	1.80	1.33	1.14	1.28
Ramachandran plot, most favored region (%)	94.2^b^	98.3^b^	97.6^b^	98.0^b^	98.3^b^
PDB code	2WO5	2WNN	2WNQ	2WNZ	2WPB

a Two additional molecules of noncovalently bound pyruvate are observed in subunits C and D.

b In all structures, 100% of residues lie within favored regions of the Ramachandran plot.
