# Supplementary material for: Structural Insights into Substrate Specificity in Variants of N-Acetylneuraminic Acid Lyase Produced by Directed Evolution
Source: J Mol Biol. 2010 Nov 19;404(1):56–69. doi: 10.1016/j.jmb.2010.08.008 (PMC3014015; doi:10.1016/j.jmb.2010.08.008)
Supplement: Table S1 — Interactions between THB and the enzyme active sites of each subunit. [file mmc1.doc]

**Table S1 Interactions between THB and the enzyme active sites of each subunit.**

H-bond interactions and hydrophobic contacts between the ligand THB (numbered as shown in Fig. 1) and the protein in each subunit as determined by LIGPLOT (Wallace *et al*, (1995) *Prot. Eng*. **8**, 127-134). In subunit B, ligand atom O5 forms an H-bond network with Tyr137 and Thr167 via a water molecule (HOH-2068). In this case the first distance is the O5 to water distance and the second is the water to protein ligand distance. In the hydrophobic contacts table, the figures shown in bold are those recognized as a contact by LIGPLOT.

H-bonds

|  |  | Distance (Å) | | | |
| --- | --- | --- | --- | --- | --- |
| Ligand atom | Protein atom | Subunit A | Subunit B | Subunit C | Subunit D |
| O4 | Ser208-N | 3.06 | 2.81 | 2.77 | 2.84 |
|  | Ser208-OG | 3.24 | 3.22 | 3.07 | 3.04 |
| O3 | HOH(2068)-  Tyr137-OH |  | 3.02-2.55 |  |  |
|  | HOH(2068)-Thr167-OG1 |  | 3.02-3.15 |  |  |
|  | Asp191-N |  |  | 3.33 |  |
| O2 | Asp191-N |  | 2.72 |  |  |
|  | Ser208-OG |  |  | 3.02 |  |
|  | HOH(2135) |  |  | 3.26 |  |
|  | HOH(2135) |  |  | 2.77 |  |

**Hydrophobic contacts**

|  |  | Distance (Å) | | | |
| --- | --- | --- | --- | --- | --- |
| Ligand atom | Protein atom | Subunit A | Subunit B | Subunit C | Subunit D |
| O4 | Gly189-O | **3.74** | **3.65** | **3.89** | **3.81** |
|  | Asp191-OD1 | **3.40** | **2.80** | **3.12** | **2.95** |
|  | Asp191-OD2 | **3.55** | **3.28** | **3.49** | **3.34** |
|  | Asp191-CG | **3.65** | **3.22** | **3.50** | **3.36** |
|  | Gly207-C | **3.84** | **3.54** | **3.58** | **3.65** |
|  | Gly207-CA | **3.62** | **3.29** | **3.42** | **3.46** |
|  | Ser208-CA | 4.0 | **3.83** | **3.72** | **3.76** |
|  | Ser208-CB | **3.65** | **3.70** | **3.51** | **3.48** |
| C4 | Pyr-C3 | **3.64** | **3.44** | **3.61** | **3.60** |
|  | Gly189-O | **3.46** | **3.69** | **3.22** | **3.70** |
|  | Ser208-N | 4.3 | **3.65** | 4.0 | **3.83** |
|  | Ser208-OG | 4.2 | **3.67** | 4.1 | **3.73** |
| O3 | Gly189-O | **2.75** | **3.56** | **2.82** | **2.74** |
|  | Gly189-C | **3.71** | 4.6 | **3.80** | **3.79** |
|  | Thr167-CG2 | 4.7 | **3.77** | 5.1 | 4.9 |
|  | Tyr190-CA | **3.32** | 5.1 | **3.37** | **3.59** |
|  | Tyr190-C | 4.0 | 6.0 | **3.88** | 4.2 |
|  | Tyr190-CD1 | **3.34** | 4.3 | **3.41** | **3.64** |
|  | Asp191-N | **3.51** | 5.7 | 3.3 | **3.73** |
|  | Asp191-OD1 | 3.9 | 5.5 | **3.57** | **3.73** |
| C3 | Gly198-O | **3.53** | **3.70** | **3.36** | **3.61** |
| O2 | Gly189-O | 5.9 | **3.01** | 5.6 | 6.0 |
|  | Asp191-OD1 | 6.3 | **2.68** | 5.6 | 6.2 |
|  | Asp191-OD2 | 6.0 | **3.66** | 5.4 | 6.1 |
|  | Asp191-CG | 6.2 | **2.96** | 5.6 | 6.2 |
|  | Asp191-CB | 6.9 | **3.36** | 6.4 | 7.0 |
|  | Asp191-CA | 7.6 | **3.61** | 7.2 | 7.7 |
|  | Tyr190-C | 7.5 | **3.59** | 7.3 | 7.7 |
|  | Tyr190-CA | 7.0 | **3.52** | 6.9 | 7.2 |
|  | Ser208-CB | 4.6 | 5.6 | **3.73** | 4.2 |
|  | Ser208-OG | **3.85** | 4.7 | 3.0 | **3.66** |
| C2 | Gly189-O | 4.9 | **3.38** | 4.8 | 5.0 |
|  | Tyr190-CA | 5.6 | **3.88** | 5.7 | 5.9 |
|  | Tyr190-CD1 | 4.7 | **3.62** | 4.9 | 5.1 |
|  | Ser208-OG | 4.3 | 4.9 | **3.90** | **3.87** |
|  | Asp191-N | 5.4 | **3.79** | 5.4 | 5.6 |
| O1 | Asp191-N | 6.9 | **3.82** | 7.4 | 6.8 |
| C1 | Tyr190-CD1 | 4.0 | **3.79** | 4.5 | 4.3 |
| N | Tyr190-CD1 | **3.73** | 4.2 | 4.1 | 4.0 |
| CAC (syn) | Tyr190-CE1 | **3.85** | 5.1 | 4.0 | 4.0 |
|  | Tyr190-CD1 | **3.86** | 5.0 | 4.5 | 3.9 |
| CAB (syn) | Asn192-OD1 | **3.70** | 5.2 | 5.6 | 6.1 |
|  | Asn192-CG | **3.64** | 5.1 | 5.7 | 5.8 |
|  | Asn192-ND2 | **2.86** | 4.3 | 4.9 | 4.9 |
|  | Tyr190-CE1 | 4.0 | 4.4 | **3.77** | 5.1 |
|  | Ile247-CD1 | 4.2 | 4.3 | 5.2 | **3.83** |
| CAA (syn terminal methyl) | Asn192-ND2 | **3.30** | 5.4 | 5.9 | 5.6 |
| CAE (anti terminal methyl) | Asp191-CB | 5.3 | 9.0 | **3.78** | **3.87** |
|  | Ile243-CG2 | **3.41** | 8.9 | 4.1 | **3.65** |
|  | Ile243-CD1 | 4.4 | 10.0 | 4.0 | **3.66** |
|  | Val251-CG2 | **3.77** | 4.6 | 5.3 | 5.2 |
|  | Ile243-CG1 | 4.1 | 9.6 | 4.3 | **3.52** |
|  | Thr209-CG2 | 4.7 | 8.2 | 4.0 | **3.08** |
| CAF (anti) | Ile243-CG2 | 4.3 | 8.0 | 4.2 | **3.67** |
